# Serum phosphate levels are related to all-cause, cardiovascular and COPD mortality in men

**DOI:** 10.1007/s10654-018-0407-7

**Published:** 2018-05-15

**Authors:** Natalia Campos-Obando, Lies Lahousse, Guy Brusselle, Bruno H. Stricker, Albert Hofman, Oscar H. Franco, André G. Uitterlinden, M. Carola Zillikens

**Affiliations:** 1 Department of Internal Medicine, Erasmus MC, PO Box 2040, 3000 CA Rotterdam, The Netherlands; 2 Department of Epidemiology, Erasmus MC, 3000 CA Rotterdam, The Netherlands; 3 Department of Respiratory Medicine, Ghent University Hospital, 9000 Ghent, Belgium; 4 Department of Respiratory Medicine, Erasmus MC, 3000 CA Rotterdam, The Netherlands

**Keywords:** Phosphate, Phosphotoxicity, Mortality, COPD, Emphysema

## Abstract

Hyperphosphatemia has been associated with increased mortality in chronic kidney disease but the nature of such a relation in the general population is unclear. To investigate the association between phosphate (P) levels and all-cause and cause-specific mortality, we assessed two cohorts from the Rotterdam Study, with follow-up of 14.5 (RS-I) and 10.9 (RS-II) years until January 2012 with availability of fasting phosphate levels. Deaths were classified according to International Classification of Diseases into 7 groups: cardiovascular, cancer, infections, external, dementia, chronic lung diseases and other causes. Sex-stratified Weibull and competing-risks models were adjusted for age, BMI and smoking. Hazard ratios are expressed per 1 mg/dL increase in phosphate levels. The total number of participants included 3731 (RS-I, 2154 women) and 2494 (RS-II, 1361 women) subjects. The main outcome measures were all-cause and cause-specific mortality. A significant positive association was found between phosphate and all-cause mortality in men (pooled HR (95% CI): 1.46 (1.26–1.69)) but not in women (0.90 (0.77–1.05)). In men, higher phosphate increased the risk for cardiovascular mortality (1.66 (1.29–2.14)), other causes (1.67 (1.16–2.40)) and chronic lung disease mortality (1.94 (1.02–3.72)), the latter driven by mortality due to chronic obstructive pulmonary disease (COPD) (4.44 (2.08–9.49)). No relations were found for mortality due to infections, cancer, dementia or external causes. In conclusion, serum P is associated with increased all-cause, cardiovascular and COPD mortality in men but not women. The association with COPD mortality is novel and needs further research on underlying mechanisms.

**Electronic supplementary material:**

The online version of this article (10.1007/s10654-018-0407-7) contains supplementary material, which is available to authorized users.

## Introduction

Phosphorus is the sixth most common element in the human body and the second mineral in abundance [1]. It plays an important structural role in hard tissues, such as bone, and exerts critical regulatory roles in metabolic and signaling pathways [1].

The majority of phosphorus is stored in bone (85%) where it is complexed with calcium in the form of hydroxyapatite, whereas 15% of phosphorus is located in the intracellular compartment while less than 1% is present in extracellular fluids. In blood, phosphorus exists in two main forms: a) an organic form bound to proteins (70%), b) an ionized form (30%), known as inorganic phosphorus, or *phosphate*, that circulates freely [1].

Traditionally, phosphate homeostatic mechanisms have been ascribed to the actions of parathyroid hormone (PTH) and 1,25-dihydroxyvitamin D3 (1,25(OH)_2_D_3_) [1, 2]. Recently, an equally important new axis of phosphate regulators was discovered [2, 3], composed of the so-called *phosphatonins:* fibroblast growth factor 23 (FGF23), synthesized mainly in osteocytes, and its co-receptor α-Klotho [3, 4]. The FGF23/α-Klotho axis increases Purinary excretion [5].

Monogenic disorders causing extreme phosphate concentrations are associated with rickets in severe hypophosphatemia and calcinosis in severe hyperphosphatemia [5]. Recently, milder hyperphosphatemia was shown to increase cardiovascular mortality in chronic kidney disease (CKD) [6]. Subsequently, this association was reported also in non-CKD population [7–10]. Interestingly, sex differences have been described with associations found in men but not women for all-cause mortality and subclinical atherosclerosis [9]; the underlying reasons are not understood. In addition to serum phosphate levels (P), high P intake has recently been found to increase mortality [11].

The objectives of this study were to assess the association of P with all-cause and, in detail, cause-specific mortality within two cohorts of the population-based Rotterdam Study, and to test for potential sex differences in these associations.

## Materials and methods

### Study population

**Fig. 1:**
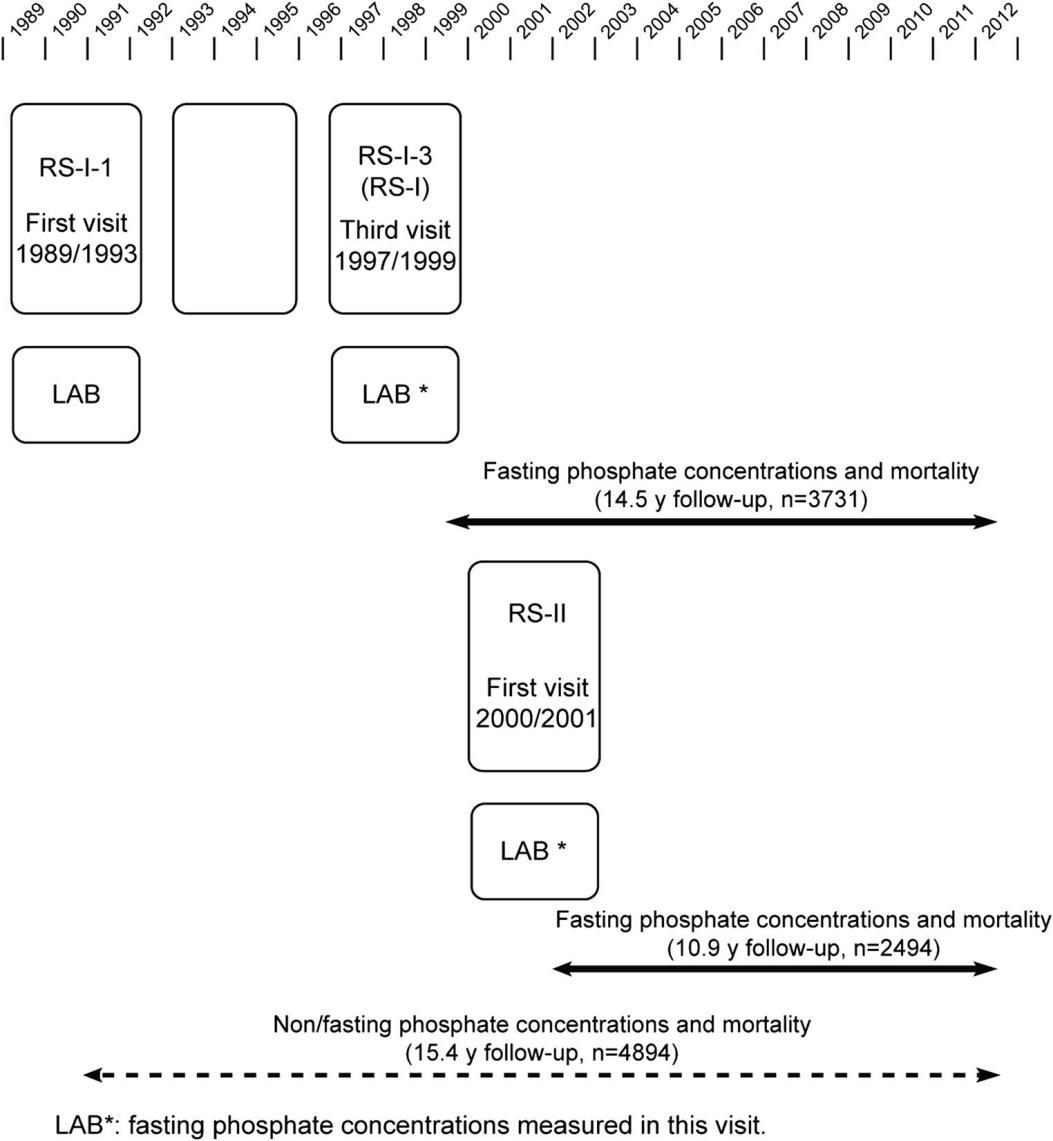
Flowchart for time line, design and sample size for the analyses

The Rotterdam Study is a prospective study of men and women designed to investigate the incidence and determinants of chronic disabling diseases. Rationale and design has been described elsewhere [12]. This research was performed in two cohorts within the Rotterdam Study, the Rotterdam Study I cohort (RS-I), initiated in 1990 in 7983 subjects, and the Rotterdam Study II cohort (RS-II) initiated in 2000 in 3011 subjects. All participants were 55 years or more at recruitment and have been assessed at baseline and through several follow-up visits. P was measured in the non-fasting state at baseline visit of RS-I (referred to as RS-I-1) and in the fasting state at the second follow-up visit of RS-I (RS-I-3, referred to as RS-I) and the baseline visit of RS-II (Fig. 1). The fasting state may modify the association between P and mortality [10]. Therefore, our main analysis was based on data from RS-I-3 and RS-II because P was assessed in the fasting state; subsequently we checked if the observed results followed similar patterns in RS-I-1, where non-fasting samples are available. A total of 3731 participants from RS-I and 2494 from RS-II were included for these analyses, all of them with signed informed consent and available phosphate levels. The Rotterdam Study was approved by the institutional review board (Medical Ethics Committee) of the Erasmus Medical Center and by the review board of the Netherlands Ministry of Health, Welfare and Sports. The approval has been renewed every 5 years.

### Laboratory measurements

The amount of phosphorus determined in blood corresponds to the inorganic fraction, or *phosphate* (mg/dL), assessed with a method based on the formation of ammonium phosphomolybdate [1].

Total calcium determination (mg/dL) was done through a colorimetric o-cresolphthalein complex one method (Merck Diagnostica, Amsterdam, The Netherlands, for RS-I-1; and Roche, Mannheim, Germany, for RS-I and RS-II). Levels of 25-hydroxyvitamin D (nmol/L) were determined through an electrochemiluminescence immunoassay. We applied cosinor regressions to adjust 25-hydroxyvitamin D for season and year. After testing for seasonality applying the dickey fuller test, we proceeded to perform a time transformation on sine and cosine terms (sin(2*pi*time/12)). Afterwards, we proceeded to regress the serum vitamin D levels on those terms to get the mesor, that is, the mean value of the cosinor regression. We then computed the difference between the mean of each season and the mesor, and adjusted every individual value accordingly [13, 14]. Levels of 1,25-dihydroxyvitamin D_3_ were assessed in a subset of participants from RS-I-1 through ^125^I-radioimmunoassay (IDS, Boldon, UK). Creatinine was determined through a sarcosine-based colorimetric assay and standardized against isotope dilution mass spectrometry (ID-MS). Cystatin C was assessed through particle enhanced immunoturbidimetric assay. C-reactive protein (CRP) levels were measured through an agglutination method with antibodies. Magnesium (Mg) levels were determined with a colorimetric method based on xylidyl blue. Glucose and cholesterol levels were determined by standard enzymatic methods [12].

### Covariates

We assessed the distribution of potential confounders across P quintiles, such as age, body mass index (BMI), smoking status, calcium, 25-hydroxyvitamin D levels, creatinine, estimated glomerular filtration rate (eGFR), C-reactive protein (CRP), glucose, magnesium, total cholesterol to HDL cholesterol ratio and prevalent diabetes mellitus and cardiovascular disease (CVD). BMI, smoking status, prevalent diabetes mellitus and prevalent CVD were assessed as previously described [12]. The diagnoses of prevalent and incident chronic obstructive pulmonary disease (COPD) cases was based on an obstructive pre-bronchodilator spirometry (FEV_1_/FVC < 0.7), according to GOLD guidelines [15]. P intake at baseline visit (RS-I-1) was collected in a subsample of participants through a validated semiquantitative food frequency questionnaire. The Chronic Kidney Disease Epidemiology Collaboration equations based on creatinine [16, 17] were applied to estimate eGFR (mL/min). Additionally, cystatin C-based eGFR was estimated for subjects with creatinine-based eGFR less than 60 mL/min, as previously recommended [16].

### Assessment of all-cause and cause-specific mortality

Information on vital status is obtained continuously from the municipal authorities in Rotterdam. The cohorts are monitored for mortality through computerized linkage of the study database to medical files of general practitioners. Two research physicians independently coded the mortality events according to ICD-10. Medical specialists in the respective field reviewed and confirmed the diagnosis. Information on cause-specific mortality was available until January, 2012.

Different causes of mortality were recorded according to ICD-10 codes and firstly grouped into cardiovascular diseases (CVD), cancer and other causes. To perform comprehensive analyses, the group of other causes was further categorized into external causes, dementia, infections, chronic lung disease and other causes in the strict sense, as previously described [18].

### Statistical analysis

Subjects with fasting P measurements from RS-I and RS-II were analyzed separately and in a meta-analysis. Additionally, we analyzed subjects with non-fasting P from RS-I-1.

Due to sex differences in P [19] and in its association with health outcomes [9], we built sex-stratified analyses. We compared the distribution of potential confounding factors applying age-adjusted tests of trend across P quintiles. We estimated P levels across smoking categories applying ANOVA and post hoc (Tukey’s) tests. Initially, the association of P with mortality was assessed through Cox models, testing the proportionality assumption of the hazards via the Schoenfeld residuals test. All significant HRs from Cox’ models were found to be constant over follow-up time; therefore, we found no evidence for a time-dependent effect of P levels on mortality. In a second step, we compared the semi-parametric Cox model with parametric models, and found that Weibull regression models—albeit with highly similar results to Cox regressions—provide better statistical fit to the data than Cox models. Weibull models provided also better fit than the rest of parametric models. We applied Cox-Snell residuals graphs and Akaike (AIC) and Bayesian information criteria (BIC) to compare among models, as previously recommended [20]. Models with lower AIC and BIC correspond to a better fit. Therefore, the results reported in this manuscript correspond to Weibull regression models. Finally, we also performed competing-risks regressions models which allow for informative censoring due to the multiple possible causes of death [21]; these models provide an estimate of the effect of the exposure on the probability of developing the outcome over time [22].

Hazard ratios (HRs) are expressed per increase in 1 mg/dL (0.32 mmol/L) of P or in quintiles; the latter were built to explore a potential dose–effect relationship between phosphate levels and mortality.

The analysis time was set at the date of blood drawing. Subjects were followed until the first of the following events happened: death, lost to follow-up, or censoring by 1st January, 2012.

Adjustments were made firstly for age, BMI and smoking because they are related to mortality and P; subsequently other covariates that have been associated with mortality were added to the model and retained if they changed the beta estimate more than 10%, including eGFR, glucose, hsCRP, Mg levels, cholesterol to HDL cholesterol ratio, calcium, 25-hydroxyvitamin D and prevalent cardiovascular disease.

Results from RS-I and RS-II were meta-analyzed using fixed-effect model.

Primary analyses were done with subjects with complete information on covariates. Subsequently, missing values were imputed via multiple imputation with chained equations, allowing missingness at random. We followed specific guidelines for imputation for survival analysis.

### Sensitivity analyses

We repeated analyses including only subjects with normal P (2.5–4.5 mg/dL; 0.81–1.45 mmol/L). We further adjusted the analyses for phosphate dietary intake and 1,25-dihydroxyvitamin D_3_ levels in a subset of participants from RS-I-1 (n = 4046).

Additionally, we performed stratified analyses according to smoking categories.

We used SPSS (version 21.0, Armonk, NY: IBM Corp), Stata (version 13, College Station TX: Stata Corp LP) and Comprehensive Meta-Analysis (version 2.2, Biostat, Englewood, NJ). A two-sided *p* <0.05 was considered significant.

## Results

### Serum phosphate correlates

A general descriptive summary of main continuous covariates is depicted in Table 1. The distribution of relevant covariates and risk factors across even quintiles of P for RS-I and RS-II is depicted in Table 2. P was higher in women than men in both cohorts (*p*_difference_ < 0.001). P levels were different across smoking categories in both sexes and cohorts (ANOVA *p* < 0.001); this difference was due to higher P in current smokers (Tukey’s tests > 0.05 between former and never smokers).

**Table 1:** General characteristics of subjects in RS-I and RS-II with serum phosphate levels, BMI and smoking information available, stratified by sex

	Men			Women		
	Mean (SD)	Minimum	Maximum	Mean (SD)	Minimum	Maximum
	(n: 1577)			(n: 2154)		
(I) RS-I						
Age (year)	71.8 (6.53)	61.4	96.7	72.5 (7.06)	61.4	100.9
BMI (kg/m^2^)	26.3 (3.18)	17.6	41.1	27.3 (4.37)	15.2	47.9
Calcium (mg/dL)	9.65 (0.39)	6.26	11.6	9.79 (0.41)	6.98	12.9
Phosphate (mg/dL)	3.15 (0.44)	1.91	7.62	3.62 (0.43)	2.28	5.25
25(OH)D (nmol/L)	61.4 (25.5)	8.99	173.8	47.9 (22.5)	5.14	134.4
CRP (mg/L)	4.24 (7.22)	0.20	115.0	3.93 (6.66)	0.20	145.0
Glucose (mmol/L)	6.06 (1.62)	4.10	20.5	5.87 (1.46)	1.60	19.5
Creatinine (μmol/L)	92.4 (33.6)	43.0	1107.0	72.1 (14.8)	34.0	263.0
eGFR (mL/min)	73.8 (14.4)	3.55	108.8	73.8 (13.9)	14.9	113.7
Mg (mmol/L)	0.85 (0.06)	0.60	1.13	0.85 (0.06)	0.58	1.17
Chol to HDL ratio	4.69 (1.32)	1.52	10.2	4.30 (1.30)	1.19	14.1

	Men (n: 1133)			Women (n: 1361)		
(II) RS-II						
Age (year)	64.3 (7.48)	55.1	93.9	64.9 (8.17)	55.1	95.3
BMI (kg/m^2^)	26.9 (3.36)	16.8	40.5	27.4 (4.46)	15.9	50.5
Calcium (mg/dL)	9.57 (0.34)	8.58	11.8	9.68 (0.34)	8.70	11.3
Phosphate (mg/dL)	3.09 (0.44)	1.39	4.66	3.54 (0.44)	1.82	5.12
25(OH)D (nmol/L)	65.7 (27.9)	0.25	175.0	58.9 (27.5)	5.84	162.5
CRP (mg/L)	2.37 (4.60)	0.30	51.8	2.33 (4.16)	0.00	65.5
Glucose (mmol/L)	6.17 (1.78)	3.90	22.1	5.87 (1.47)	3.80	25.9
Creatinine (μmol/L)	87.8 (18.7)	53.0	349.0	69.2 (11.8)	40.0	165.0
eGFR (mL/min)	80.9 (13.9)	14.0	111.6	80.6 (13.7)	26.8	108.4
Mg (mmol/L)	0.83 (0.06)	0.34	1.02	0.83 (0.06)	0.43	1.06
Chol to HDL ratio	4.77 (1.34)	1.83	12.4	4.23 (1.22)	1.52	11.1

*BMI* body mass index, *25(OH)D* 25-hydroxyvitamin D levels, *CRP* C-reactive protein, *eGFR* estimated glomerular filtration rate, *Mg* magnesium, *Chol to HDL ratio* total cholesterol to HDL cholesterol ratio

Conversion to SI Units: to convert 25-hydroxyvitamin D levels to ng/mL multiply by 0.4; to convert glucose to mg/dL multiply by 18.02; to convert creatinine to mg/dL multiply by 0.011; to convert magnesium to mg/dL multiply by 2.43

**Table 2:** General characteristics of subjects in RS-I and RS-II according to quintiles of fasting phosphate levels

	Men						Women					
	Phosphate in quintiles						Phosphate in quintiles					
	1	2	3	4	5	p*	1	2	3	4	5	p*
(I) RS-I												
N (mg/dL)	315 (2.56)	315 (2.92)	316 (3.15)	315 (3.37)	316 (3.77)		431 (3.02)	431 (3.40)	431 (3.62)	431 (3.83)	430 (4.21)	
Age (year)	71.6	72.4	71.4	71.9	71.8	0.968	73.0	72.3	72.8	72.4	71.9	**0.049**
BMI (kg/m^2^)	26.7	26.4	26.2	26.2	26.0	**0.005**	29.0	27.5	27.2	26.7	25.9	**< 0.001**
Ever smoke (%)	90%	87%	92%	92%	95%	**0.008**	47%	47%	50%	53%	51%	0.091
Calcium (mg/dL)	9.59	9.66	9.62	9.66	9.72	**< 0.001**	9.77	9.80	9.76	9.79	9.84	**0.026**
25 (OH) D (nmol/L)	62.8	62.6	62.7	59.1	59.9	**0.034**	45.4	48.9	46.8	48.6	50.1	**0.035**
CRP (mg/L)	4.57	3.62	4.15	3.79	5.12	0.340	4.92	4.06	3.79	3.60	3.23	**< 0.001**
Glucose (mmol/L)	6.09	5.96	6.04	6.04	6.15	0.529	6.18	5.79	5.87	5.77	5.77	**< 0.001**
Prevalent DM (%)	14%	12%	13%	14%	15%	0.424	17%	10%	12%	9%	9%	**0.001**
Creatinine (μmol/L)	91.4	92.7	90.2	91.3	96.2	0.167	72.4	72.7	71.5	71.9	71.9	0.652
eGFR (mL/min)	73.9	72.0	75.0	74.2	73.7	0.432	73.1	73.1	74.2	74.1	74.4	0.356
Mg (mmol/L)	0.84	0.84	0.85	0.85	0.86	**0.002**	0.84	0.85	0.85	0.85	0.86	**< 0.001**
Chol to HDL ratio	4.75	4.93	4.75	4.56	4.47	**< 0.001**	4.41	4.42	4.33	4.17	4.18	**< 0.001**
Prevalent CVD (%)	7%	9%	8%	7%	10%	0.221	4%	2%	2%	4%	3%	0.712
(II) RS-II												
N (mg/dL)	226 (2.49)	227 (2.86)	226 (3.07)	227 (3.31)	227 (3.71)		272 (2.92)	272 (3.32)	272 (3.54)	272 (3.77)	273 (4.14)	
Age (year)	63.8	64.4	65.0	64.7	63.8	0.884	65.4	66.2	64.4	65.2	63.1	**< 0.001**
BMI (kg/m^2^)	27.1	26.7	26.8	26.7	27.3	0.482	29.1	27.9	27.4	26.8	26.0	**< 0.001**
Ever smoke (%)	86%	81%	80%	87%	89%	0.142	57%	62%	57%	58%	63%	0.642
Calcium (mg/dL)	9.50	9.59	9.53	9.58	9.64	**< 0.001**	9.64	9.66	9.70	9.68	9.75	**0.001**
25 (OH) D (nmol/L)	66.6	68.0	65.1	65.7	62.8	0.103	57.1	57.2	58.3	58.9	63.2	0.071
CRP (mg/L)	2.41	2.22	2.42	1.88	2.93	0.479	2.82	2.54	1.92	2.34	2.01	**0.037**
Glucose (mmol/L)	6.08	5.98	6.24	6.06	6.50	**0.013**	6.10	5.84	5.79	5.78	5.84	**0.049**
Prevalent DM (%)	11%	9%	15%	11%	21%	**0.002**	12%	9%	10%	9%	7%	0.164
Creatinine (μmol/L)	87.6	87.4	88.7	86.8	88.2	0.915	69.5	70.0	68.4	69.2	68.9	0.923
eGFR (mL/min)	80.9	81.3	79.2	81.3	81.7	0.475	79.7	79.1	81.6	80.5	81.8	0.691
Mg (mmol/L)	0.83	0.83	0.83	0.83	0.84	0.290	0.82	0.83	0.83	0.83	0.84	**< 0.001**
Chol to HDL ratio	4.93	4.71	4.62	4.66	4.93	0.906	4.35	4.20	4.31	4.15	4.12	**0.042**
Prevalent CVD (%)	8%	11%	13%	9%	13%	0.351	3%	3%	3%	3%	1%	0.608

Statistically significant* p*-values (<0.05) are highlighted in bold font

*P values corresponds to age-adjusted significance of trend across quintiles

*BMI* body mass index, *25(OH)D* 25-hydroxyvitamin D levels, *CRP* C-reactive protein, *prevalent DM* prevalent diabetes mellitus, *eGFR* estimated glomerular filtration rate, *Mg* magnesium, *Chol to HDL ratio* total cholesterol to HDL cholesterol ratio, *prevalent CVD* prevalent cardiovascular disease

Conversion to SI Units: to convert 25-hydroxyvitamin D levels to ng/mL multiply by 0.4; to convert glucose to mg/dL multiply by 18.02; to convert creatinine to mg/dL multiply by 0.011; to convert magnesium to mg/dL multiply by 2.43

P was within normal range in 95.5 and 94.9% of participants in the fasting state (RS-I and RS-II, respectively) and in 89.7% of participants in the non-fasting state (RS-I-1).

### Serum phosphate and all-cause mortality

**Fig. 2:**
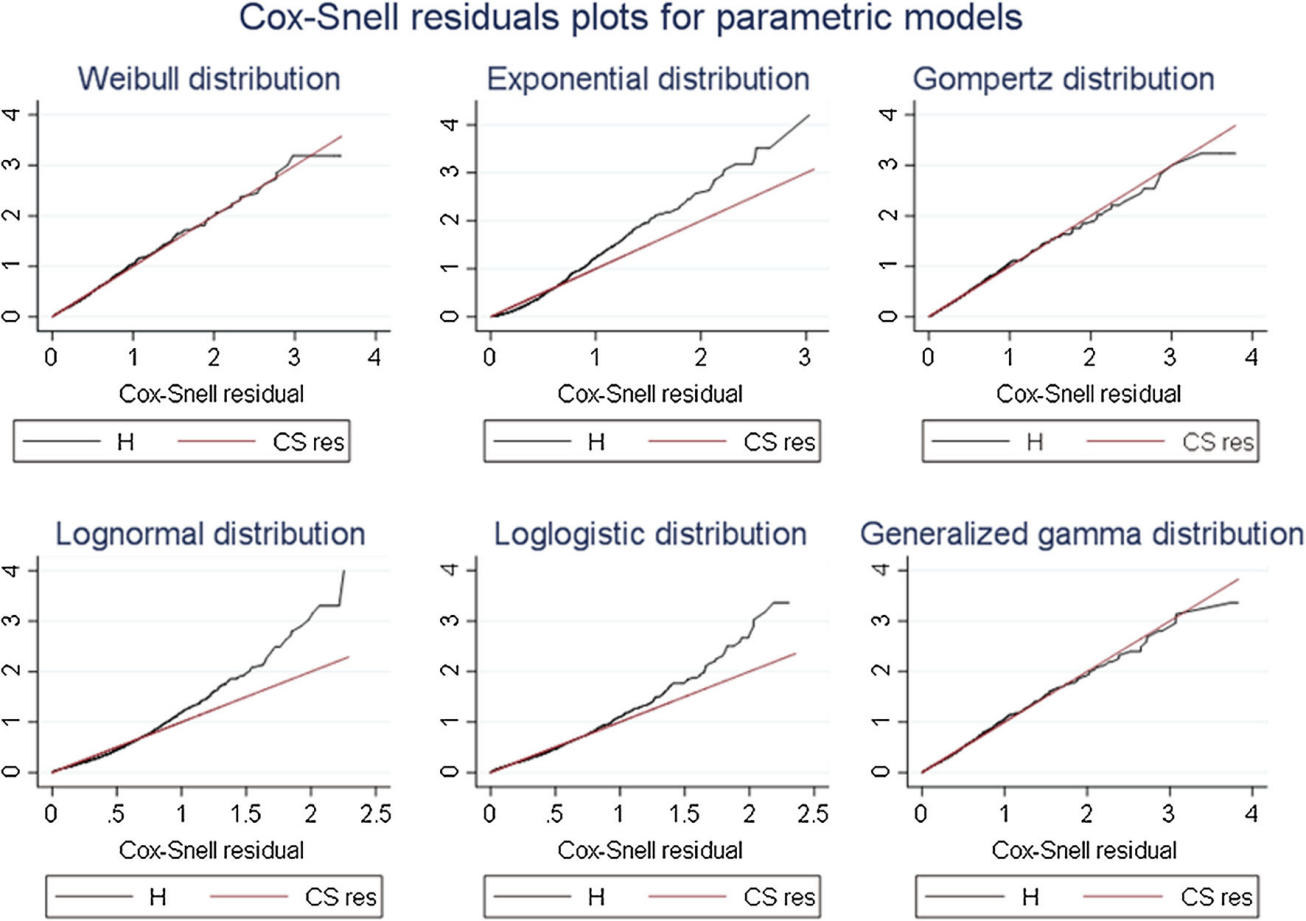
Cox-Snell residuals plot for parametric models in the association between serum phosphate levels and all-cause mortality in men

During 14.5 year (median) and 10.9 year (mean) follow-up a total of 1631 and 469 fatal events occurred in RS-I and RS-II, respectively. We found a significant interaction between P and sex for all-cause mortality in RS-I (*p*_interaction_ < 0.001) and performed sex-stratified analyses. The results for the comparison of goodness-of-fit between parametric models and the semiparametric Cox model are displayed in Supplementary Table 1 (AIC and BIC criteria) and in Fig. 2 (Cox-Snell residuals plot). Both methods showed that Weibull models provide a better fit to our data among the parametric and semiparametric models.

The associations between P and all-cause mortality are depicted in Table 3. Results from RS-I and RS-II were meta-analyzed (pooled HR (95% CI)). A significant association between P and all-cause mortality was found in men (1.46 (1.26–1.69)) but not in women (0.90 (0.77–1.05)). Adjustments in a full model composed of age, BMI, smoking, prevalent cardiovascular disease and levels of calcium, 25-hydroxyvitamin D, eGFR, CRP, Mg, glucose and total cholesterol to HDL cholesterol ratio levels did not substantially modify results (men: 1.49 (1.27–1.74); women: 0.92 (0.79–1.07)).

**Table 3:** Serum phosphate levels and all-cause mortality in RS-I and RS-II, adjusted for age, BMI and smoking, follow-up until year 2012

	Men			Women		
	n_o_ events	HR* (95% CI)	*p*	n_o_ events	HR* (95% CI)	*p*
RS-I	810/1577	**1.58** (1.34–1.87)	< 0.001	821/2154	0.85 (0.71–1.00)	0.056
RS-II	262/1133	1.14 (0.85–1.53)	0.378	207/1361	1.14 (0.81–1.60)	0.439
Studies combined^†^	1072/2710	1.46 (1.26–1.69)	< 0.001	1028/3515	0.90 (0.77–1.05)	0.176

Statistically significant* p*-values (<0.05) are highlighted in bold font

*Hazard ratios from Weibull models, expressed per 1 mg/dL (0.32 mmol/L) increase in phosphate levels

^†^Studies combined from meta-analyses using fixed-effect models

Similarly, results from RS-I-1 with non-fasting phosphate showed a significant association of phosphate with all-cause mortality in men (1.12 (1.02–1.23); n_o_ events = 1389), but not in women (0.99 (0.91–1.08); n_o_ events = 1779).

To explore whether there was a dose–response pattern in the association we found in men, we analyzed P in even quintiles and all-cause mortality in RS-I, the cohort with most events, (Table 4) and set the first quintile (lowest) as reference. We observed a significant trend for increasing P and mortality (*p*_trend_ < 0.001) with significant HRs for the fourth (1.35 (1.08–1.69)) and fifth quintile (1.49 (1.19–1.86)) compared with the first quintile.

**Table 4:** Serum phosphate levels in quintiles and all-cause mortality in men from RS-I, adjusted for age, BMI and smoking, follow-up until year 2012

Quintile	Phosphate concentrations mean (range)*	n_o_. events/n_o_. risk	HR^†^ (95% CI)	*p*
1	2.56 (1.91–2.81)	139/315	1 (reference)	
2	2.92 (2.81–3.02)	154/315	1.09 (0.87–1.38)	0.439
3	3.15 (3.02–3.27)	154/316	1.05 (0.83–1.33)	0.660
4	3.37 (3.27–3.49)	172/315	**1.35** (1.08–1.69)	0.008
5	3.77 (3.52–7.62)	191/316	**1.49** (1.19–1.86)	< 0.001
*p*_trend_				< 0.001

Statistically significant* p*-values (<0.05) are highlighted in bold font

*Phosphate levels in mg/dL

^†^Hazard ratios from Weibull models; first quintile was set as reference

### Sensitivity analyses

Results after excluding subjects with abnormal P were similar to the unrestricted analyses (men: 1.44 (1.21–1.70); women: 0.87 (0.74–1.03)). Adjustments for phosphate and energy intake in men from RS-I-1 did not modify the results between non-fasting phosphate and all-cause mortality (1.13 (1.02–1.24); n_o_ events = 1117). Further adjustments for 1,25 dihydroxyvitamin D_3_ levels in a subset from RS-I-1 did not modify results (data not shown).

### Serum phosphate and cause-specific mortality in men

We did not observe associations between P and cause-specific mortality in women (data not shown). In contrast, the pooled results in men (Table 5) showed a significant positive relation between P and CVD mortality (1.66 (1.29–2.14)). Exclusion of male subjects with prevalent CVD disease yielded similar results (1.69 (1.28–2.23)).

**Table 5:** Serum phosphate levels and cause-specific mortality in men from RS-I and RS-II, adjusted for age, BMI and smoking, follow-up until year 2012

	Individual cohorts				Studies combined	
	Cohort	n	HR^*^ (95% CI)	*p*	HR^†^ (95% CI)	*p*
CVD	RS-I	266	**1.80** (1.35–2.39)	< 0.001	**1.66** (1.29–2.14)	< 0.001
	RS-II	77	1.25 (0.73–2.15)	0.412		
Cancer	RS-I	243	**1.41** (1.04–1.90)	0.025	1.23 (0.95–1.58)	0.112
	RS-II	98	0.88 (0.55–1.40)	0.586		
External	RS-I	18	1.58 (0.50–5.02)	0.439	0.94 (0.36–2.46)	0.902
	RS-II	9	0.29 (0.05–1.62)	0.159		
Infectious	RS-I	56	1.02 (0.53–1.98)	0.943	0.97 (0.53–1.80)	0.929
	RS-II	9	0.71 (0.13–3.84)	0.691		
Dementia	RS-I	52	1.83 (0.93–3.60)	0.081	1.70 (0.92–3.15)	0.092
	RS-II	13	1.18 (0.26–5.37)	0.826		
Lung	RS-I	42	2.07 (0.97–4.42)	0.058	**1.94** (1.02–3.72)	0.044
	RS-II	15	1.64 (0.47–5.72)	0.441		
Other	RS-I	133	**1.58** (1.04–2.41)	0.032	**1.67** (1.16–2.40)	0.006
	RS-II	40	1.98 (0.96–4.11)	0.066		

Statistically significant* p*-values (<0.05) are highlighted in bold font

*Hazard ratios from Weibull models, expressed per 1 mg/dL (0.32 mmol/L) increase in phosphate levels

^†^Studies combined from meta-analyses using fixed-effect models

We also found an association between higher P and chronic lung disease mortality (1.94 (1.02–3.72)). Most of these cases clustered within COPD mortality. Therefore, we further investigated such a relation (Table 6), and found a significant association (4.44 (2.08–9.49)). Most likely due to power constraints, this association was not significant in RS-II (05 cases in contrast to 28 cases in RS-I) but there was no evidence for statistical difference between both estimates (*p*_heterogeneity_ = 0.780). Further adjustments for glomerular filtration rate did not abolish the association between P and COPD mortality (4.16 (2.05–8.43)). Furthermore, the association was found to be consistent in subjects without chronic kidney disease (CKD) (6.58 (2.59–16.7)); whereas we found no association in subjects with CKD (1.14 (0.20–6.63)), although the latter analysis is constrained due to low number of events and driven only by RS-I. Non-fasting phosphate levels and COPD mortality in men from RS-I-1 also displayed a significant association (1.54 (1.05–2.27), n_o_ events = 69).

**Table 6:** Serum phosphate levels and chronic obstructive pulmonary disease (COPD) mortality in men from RS-I and RS-II, adjusted for age, BMI and smoking, follow-up until year 2012

	Individual cohorts			Studies combined	
	n	HR^*^ (95% CI)	*P*	HR^†^ (95% CI)	*p*
**RS-I**	28	**4.62** (2.06–10.3)	<0.001	**4.44** (2.08–9.49)	< 0.001
**RS-II**	05	3.29 (0.35–30.7)	0.296		

Statistically significant* p*-values (<0.05) are highlighted in bold font

*Hazard ratios from Weibull models, expressed per 1 mg/dL (0.32 mmol/L) increase in phosphate levels

^†^Studies combined from meta-analyses using fixed-effect models

P was also found to be positively associated with mortality from other causes (1.67 (1.16–2.40)).

We found no significant associations between P and death due to cancer, infections, dementia or external causes.

Results from competing-risks regression models were similar to Weibull models and showed a significant association between P and mortality due to CVD (1.50 (1.12–2.02)), other causes (1.40 (1.01–1.93)) and COPD (2.42 (1.62–3.63)); no other significant associations were found (Supplementary Tables 2 and 3).

Analyses after applying multiple imputation yielded significant associations for P and all-cause, CVD, COPD and other causes of mortality in men (data not shown). Missingness of covariates of interest was less than 6%.

### Sensitivity analyses

Results after excluding male subjects with abnormal P were similar to the unrestricted analyses (Supplementary Tables 4 and 5). Likewise, our findings remained essentially unaltered after adjustments for calcium and 25-hydroxyvitamin D levels; and were only slightly attenuated after further adjustments for levels of calcium, 25-hydroxyvitamin D and eGFR (CVD 1.65 (1.27–2.14), COPD 3.79 (1.87–7.69), other causes 1.76 (1.21–2.56)). Similar results were obtained after adjustments for cystatin-based eGFR. Additionally, the analyses after exclusion of male subjects with eGFR < 60 mL/min showed a positive association between P and mortality due to other causes (1.72 (1.13–2.61)) and COPD (6.58 (2.59–16.7)) - as previously mentioned- and a borderline association between P and CVD mortality (1.36 (1.00–1.85)).

Smoking adjustment did not attenuate the association between P and CVD or COPD mortality (data not shown). The results from the stratified analyses according to smoking categories (Supplementary Tables 6 and 7) showed that in studies combined the associations between P and all-cause and CVD mortality were in the same direction and did not show statistical evidence for a difference across categories (*p*_heterogeneity_ = 0.752 for all-cause mortality and *p*_heterogeneity_ = 0.796 for CVD mortality). The relation between P and COPD mortality in men from RS-I (RS-II excluded due to few events) was not statistically different among former and current smokers (*p*_heterogeneity_ = 0.494).

As previously mentioned, analyses in men from RS-I-1 showed that non-fasting phosphate levels were also associated with chronic lung disease mortality and COPD mortality, and these associations were not abolished after further adjustments for phosphate and energy intake: chronic lung disease mortality: 1.79 (1.19–2.68); n_o_ events = 59; COPD mortality: 1.87 (1.20–2.91), n_o_ events = 49.

## Discussion

This prospective population-based cohort study among elderly demonstrated that P was positively associated with all-cause mortality in men but not in women, supporting an effect modification by sex previously described [9]. When analyzing in detail cause-specific mortality in men, we found that this association was driven by mortality due to CVD, COPD and other causes. The association between increasing P and the composite endpoint of fatal and non-fatal CVD incidence in non-CKD population in sex-combined analyses has been reported before but is still scarce [7–9]. Our results provide evidence of an association between higher P—even within normal range—and death due to CVD in men. On the other hand, to the best of our knowledge the association we found with COPD mortality is novel. These results remained significant after adjustments for several potential confounders, were observed also after restricting the analyses to subjects with normal P and showed no heterogeneity between cohorts.

Several mechanisms have to be considered when analyzing P and mortality, including phosphate being a marker of another risk factor or through direct pathogenic pathways.

First, P levels are regulated by a complex interplay of factors that have been linked to mortality, such as 1,25-dihydroxyvitamin D_3_, PTH and FGF23. Low levels of 25-hydroxyvitamin D and 1,25-dihydroxyvitamin D_3_ have been found to be associated with increased mortality [23]. Nevertheless, the vitamin D adjustments did not modify our results.

PTH abnormalities have also been associated with mortality. Primary excess of PTH is associated with increased cardiovascular mortality [24], but in this context P tends to be low. Secondary elevations of PTH in impaired kidney function have been inconsistently associated with mortality. This compensatory mechanism in CKD is triggered when eGFR falls below 47 mL/min [25]. Although PTH levels were not available, the proportion of patients in our cohorts with eGFR below that threshold was considerably low (4% in RS-I and 2% in RS-II) suggesting that secondary hyperparathyroidism is unlikely to explain our findings. Nevertheless, PTH values seem to rise within normal range in the general population without CKD [26] at higher thresholds of decreasing eGFR (< 120 mL/min); whether increasing PTH values within normal range are associated with all-cause mortality in the long term is unclear [27].

Other important players in P homeostasis that might underlie its associations with mortality are the *phosphatonins* FGF23 and α-Klotho [3, 4]. FGF23 is synthesized mainly in osteocytes [5] and requires the presence of α-Klotho to bind to its receptor with high affinity and for signaling [28]. FGF23/α-Klotho axis decreases P through increased urinary phosphate excretion and both molecules are anti-ageing factors [5]. Primary causes of excess FGF23, such as in hereditary hypophosphatemic rickets, have been associated with cardiovascular calcification in cases of excessive phosphate treatment. Secondary FGF23 elevation occurs in CKD at earlier stages than PTH [3, 25] in response to P retention, and it has been linked to increased mortality [29, 30]. Similar to PTH, FGF23 elevations within normal range have been described at high thresholds of eGFR in population without CKD [26]; FGF23 levels have also been associated with mortality in this setting [31]. Nevertheless, FGF23 seems not to induce vascular calcification in most studies [4, 32, 33].

Recently, soluble klotho has been linked to increased mortality in CKD patients [34] although the lack of a validated assay for its measurement might be a concern for some [30].

Another potential confounder could be smoking. Similar to previous reports [7], P was found to be higher in current smokers. Although adjustments for smoking did not alter our analyses, due to heavy current and former smoking in men it is difficult to fully dissect its effects. Nevertheless, in studies combined the stratified analyses by smoking status showed that the associations between P and all-cause and CVD mortality appeared to be of the same direction and similar magnitude across smoking categories. The group of former smokers—who had similar P as never smokers—displayed the most statistically significant associations possibly due to larger number of subjects in this category. Specifically, P was related to COPD mortality comparably in current and former smokers men from RS-I but only significant in the latter group; a relation in non-smokers could not be tested due to low numbers in this subgroup. Therefore we do not anticipate that current smoking explains the association between P and COPD mortality.

Regarding direct effects, P itself is able to induce vascular calcification, a process with high resemblance to bone ossification and that increases mortality [33, 35]. Several pathways are known such as (a) differential gene expression in vascular smooth muscle cells with up-regulation of markers critical for mineralization [36]; and (b) elastin degradation, thought to be mediated by P induction of matrix metalloproteinase (MMP)-9.

The association we found between P and COPD mortality has never been described in humans before; interestingly there is additional evidence for the pathogenicity of high P stemming from rodent models with *fgf23* or *klotho* knockout. These animals display similar phenotypes characterized by severe hyperphosphatemia and features of premature aging, such as osteoporosis, ectopic calcifications, pulmonary emphysema and short life span [37–39]. Heterozygous klotho mice also display emphysematous lungs. Remarkably, a low phosphate diet is able to alleviate or rescue the phenotype -including the lung emphysema; and a high phosphate diet worsens it [40], strongly suggesting that phosphate itself accelerates ageing [41] and induces alveoli destruction, and that this process can be modified by diet manipulation [40].

A new concept of *phosphotoxicity* as a risk factor for mammalian ageing has emerged lately [3, 40] and there are concerns that increasing phosphate intake through food additives may negatively influence multiple aspects of health [42]. Indeed it has been shown that high absolute P intake was positively related to all-cause mortality -not explained by CVD mortality [11]. Recently, a healthy diet—according to the Alternate Healthy Eating Index (2010) score—was associated with lower risk of COPD in humans [43]; interestingly in men but not women this beneficial association was driven mostly by a drastic reduction in red and processed meat consumption, expected to contain high phosphate [42]. A positive relation between cured meat intake and COPD risk has previously been reported in cross-sectional (NHANES III) and prospective studies [44, 45]. Importantly, when spirometric definitions for lung volumes and COPD have been applied, cured meat intake has been shown to be negatively associated with lung function, and positively related with COPD risk [44, 46]; the latter study showed that these associations were found predominantly in men. Cured meat consumption has also been shown prospectively to increase the hospital readmission rate in COPD patients [47].

From a mechanistic point of view, previous research [48] has shown that *phosphate is able to directly induce injury in mice and human lung epithelial cells* through increased DNA oxidative stress and apoptosis; indeed phosphate medium is used experimentally to induce oxidative lung injury. Interestingly, α-Klotho exerts protective antioxidant effects against lung injury induced by P [48], hyperoxia, and acute α-Klotho deficiency [49]. These data show that lung tissue is a target for phosphotoxic insult. Remarkably, increased P intake down-regulates α-Klotho expression in rodents [41]; therefore low P diet may be a therapeutic strategy to increase Klotho [3].

A genetic variant associated with low FGF23 was found to be associated with emphysema in smokers with COPD. More studies are needed to elucidate further the underlying mechanisms, especially considering that COPD ranks high in the most common causes of death worldwide.

The reasons for the sex difference between P and mortality are not clear. Interestingly, the vascular calcification induction by P is attenuated by 17β-estradiol, suggesting a potential hormonal reason for this difference [50]. Despite the fact that menopause is characterized by low estradiol levels, hormone replacement therapy-naïve postmenopausal women with higher 17β-estradiol levels display lower coronary calcification scores than those with lower 17β-estradiol [51]. Additionally, coronary infusion of 17β-estradiol exerts vasodilation in postmenopausal women, but not men [52]. Testosterone and estradiol play important roles as P regulators [19].

Although men had a less healthy profile at baseline than women, multiple adjustments did not abolish our results. Moreover, a previous study showed that P is associated with subclinical atherosclerosis in men (but not women) without prevalent cardiovascular and cerebrovascular disease at baseline [53].

This study has several limitations. 1,25-dihydroxyvitamin D_3_ levels were available only in a subgroup. PTH and FGF23 measurements were not available and it is known that kidney function in elderly can be misclassified even by eGFR. Our findings cannot be generalized to other ethnicities other than European Caucasians. Nevertheless, there are several strengths, such as the availability of two well-characterized cohorts with long follow-up, the detailed information on cause-specific mortality and the availability of multiple potential confounders. The completeness of follow-up was high (94 and 92% in RS-I and RS-II) indicating that obtained estimates are valid.

In conclusion, we found that higher P is associated with increased all-cause mortality and cause-specific mortality due to CVD, COPD and other causes in elderly men but not in women, adding more evidence for a modification of these associations by sex. We hereby provide evidence to support that the concept of phosphotoxicity also among non-CKD general population deserves further attention and, if causally related, it occurs independently of vitamin D levels and kidney function. Our study suggests that moderation of phosphate intake might be relevant also in non-CKD population for healthy ageing. Finally, we consider that the available evidence calls for a review of the currently accepted normal range of P. Further research is needed to clarify the underlying mechanisms, especially for COPD mortality, and to elucidate the reasons for the sex difference in the association of P with mortality.

## Electronic supplementary material

Below is the link to the electronic supplementary material.

Supplementary material 1 (DOCX 41 kb)
