# Supplementary material for: Serum phosphate levels are related to all-cause, cardiovascular and COPD mortality in men
Source: Eur J Epidemiol. 2018 May 15;33(9):859–71. doi: 10.1007/s10654-018-0407-7 (PMC6133003; doi:10.1007/s10654-018-0407-7)
Supplement: Supplementary file 1 — Supplementary material 1 (DOCX 41 kb) [file 10654_2018_407_MOESM1_ESM.docx]

**SUPPLEMENTARY TABLES FOR THE MANUSCRIPT ENTITLED:**

***Serum phosphate levels are related to all-cause, cardiovascular and COPD mortality in men***

Campos-Obando N ^1^, Lahousse L ^2,3^ , Brusselle GG ^2,3,4^ , Stricker BH ^2^ , Hofman A ^2^,

Franco OH ^2^, Uitterlinden AG ^1,2^ , Zillikens MC ^1,2^

^1^ Department of Internal Medicine, Erasmus MC, 3000 CA Rotterdam; ^2^ Department of Epidemiology, Erasmus MC, 3000 CA Rotterdam; ^3^ Department of Respiratory Medicine, Ghent University Hospital; B-9000 Ghent, Belgium; ^4^ Department of Respiratory Medicine, Erasmus MC, 3000 CA Rotterdam, The Netherlands

**Short title:** Phosphate levels and mortality

**Journal name: EUROPEAN JOURNAL OF EPIDEMIOLOGY**

**Number of Supplementary tables**: 7

**Corresponding author**:

M. Carola Zillikens, MD, PhD

Request for reprints should be sent to this address: PO Box 2040 3000 CA Rotterdam, The Netherlands

E-mail: [m.c.zillikens@erasmusmc.nl](mailto:m.c.zillikens@erasmusmc.nl)

Phone: +31-10-7040704.

Fax number: +31-10-7033268.

DISCLOSURE STATEMENT: The authors have nothing to disclose.

**Supplementary Table 1.** Goodness of fit among parametric and semiparametric models for the association between serum phosphate levels and mortality in men from RS-I, according to AIC and BIC criteria:

|  | AIC | BIC |
| --- | --- | --- |
| Parametric models | | |
| Weibull | **3060.27** | **3097.81** |
| Exponential | 3246.42 | 3278.59 |
| Gompertz | 3065.36 | 3102.9 |
| Lognormal | 3194.43 | 3231.97 |
| Loglogistic | 3106.07 | 3143.62 |
| Generalized gamma | 3060.31 | 3103.22 |
| Semiparametric model | | |
| Cox | 10865.15 | 10891.96 |

AIC: Akaike information criteria; BIC: Bayesian information criteria

**Supplementary Table 2.** Competing-risk results for serum phosphate levels and cause-specific mortality in men from RS-I and RS-II, adjusted for age, BMI and smoking, follow-up until year 2012:

|  | **Individual cohorts** | | | | | **Studies combined** | | |
| --- | --- | --- | --- | --- | --- | --- | --- | --- |
|  | **Cohort** | **N** | **SHR** ^*^ **(95% CI)** | | ***p*** |  | **SHR ^*,^**^†^ **(95% CI)** | ***p*** |
| **CVD** | RS-I | 266 | **1.60** (1.14-2.24) | | 0.006 | **1.50** (1.12-2.02) 0.006 | | |
|  | RS-II | 77 | 1.24 (0.68-2.26) | | 0.488 |  |  |  |
| **Cancer** | RS-I | 243 | 1.20 (0.92-1.56) | | 0.179 | 1.10 (0.87-1.39) 0.409 | | |
|  | RS-II | 98 | 0.83 (0.51-1.34) | | 0.438 |  |  |  |
| **External** | RS-I | 18 | 1.18 (0.45-3.13) | | 0.736 | 0.73 (0.33-1.59) 0.426 | | |
|  | RS-II | 09 | 0.30 (0.08-1.11) 0.071 | | |  |  |  |
| **Infectious** | RS-I | 56 | 0.81 (0.43-1.50) | 0.497 | | 0.79 (0.44-1.41) 0.427 | | |
|  | RS-II | 09 | 0.66 (0.13-3.48) | 0.628 | |  |  |  |
| **Dementia** | RS-I | 52 | 1.31 (0.83-2.06) | 0.240 | | 1.30 (0.84-2.01) 0.235 | | |
|  | RS-II | 13 | 1.21 (0.28-5.20) | 0.797 | |  |  |  |
| **Lung** | RS-I | 42 | 1.49 (0.83-2.70) | 0.184 | | 1.48 (0.87-2.50) 0.149 | | |
|  | RS-II | 15 | 1.42 (0.43-4.64) | 0.564 | |  |  |  |
| **Other** | RS-I | 133 | 1.27 (0.88-1.84) | 0.202 | | **1.40** (1.01-1.93) 0.043 | | |
|  | RS-II | 40 | 1.91 (0.98-3.74) | 0.059 | |  |  |  |

* Subhazard ratios from competing risks models, interpreted as the relative increase in the

incidence of the event of interest per 1 mg/dL (0.32 mmol/L) increase in serum phosphate

in the presence of competing risks.

† Studies combined from meta-analyses using fixed-effect models

**Supplementary Table 3.** Competing-risks results for serum phosphate levels and COPD mortality in men from RS-I and RS-II, adjusted for age, BMI and smoking, follow-up until year 2012:

|  | **Individual cohorts** | | | **Studies combined** | | |
| --- | --- | --- | --- | --- | --- | --- |
|  | **n** | **SHR** ^*^ **(95% CI)** | ***P*** |  | **SHR ^*,^**^†^ **(95% CI)** | ***P*** |
| **RS-I** | 28 | **2.42** (1.61-3.65) | <0.001 |  | **2.42** (1.62-3.63) <0.001 | |
| **RS-II** | 05 | 2.54 (0.20-32.8) | 0.475 |  |  |  |

* Subhazard ratios from competing risks models, interpreted as the relative increase in the

incidence of the event of interest per 1 mg/dL (0.32 mmol/L) increase in serum phosphate

in the presence of competing risks.

† Studies combined from meta-analyses using fixed-effect models

**Supplementary Table 4**. Serum phosphate levels within normal range and cause-specific mortality in men from RS-I and RS-II, adjusted for age, BMI and smoking, follow-up until year 2012

|  | **Individual cohorts** | | | | | **Studies combined** | | |
| --- | --- | --- | --- | --- | --- | --- | --- | --- |
|  | **Cohort** | **N** | **HR** ^*^ **(95% CI)** | | ***p*** |  | **HR** ^†^ **(95% CI)** | ***p*** |
| **CVD** | RS-I | 249 | **1.72** (1.22-2.42) | | 0.002 | **1.60** (1.19-2.16) 0.002 | | |
|  | RS-II | 73 | 1.27 (0.68-2.37) | | 0.449 |  |  |  |
| **Cancer** | RS-I | 230 | 1.41 (0.99-2.01) | | 0.056 | 1.33 (0.98-1.80) 0.066 | | |
|  | RS-II | 82 | 1.13 (0.63-2.01) | | 0.681 |  |  |  |
| **External** | RS-I | 18 | 1.24 (0.33-4.59) | | 0.750 | 0.80 (0.25-2.58) 0.703 | | |
|  | RS-II | 8 | 0.14 (0.01-1.84) 0.134 | | |  |  |  |
| **Infectious** | RS-I | 54 | 0.61 (0.28-1.34) | 0.220 | | 0.58 (0.28-1.20) 0.142 | | |
|  | RS-II | 9 | 0.37 (0.04-.3.04) | 0.358 | |  |  |  |
| **Dementia** | RS-I | 51 | 1.61 (0.75-3.47) | 0.222 | | 1.63 (0.81-3.27) 0.171 | | |
|  | RS-II | 12 | 1.72 (0.32-9.33) | 0.530 | |  |  |  |
| **Lung** | RS-I | 39 | **2.88** (1.21-6.89) | 0.017 | | **2.31** (1.11-4.83) 0.026 | | |
|  | RS-II | 15 | 1.32 (0.33-5.32) | 0.691 | |  |  |  |
| **Other** | RS-I | 127 | 1.53 (0.94-2.50) | 0.085 | | **1.66** (1.09-2.53) 0.018 | | |
|  | RS-II | 38 | 2.11 (0.93-4.82) | 0.075 | |  |  |  |

* Hazard ratios from Weibull models, expressed per 1 mg/dL (0.32 mmol/L) increase in phosphate

levels constrained to normal values (2.5-4.5 mg/dL; 0.81-1.45 mmol/L)

† Studies combined from meta-analyses using fixed-effect models

**Supplementary Table 5**. Serum phosphate levels within normal range and chronic obstructive pulmonary disease (COPD) mortality in men from RS-I and RS-II, adjusted for age, BMI and smoking, follow-up until year 2012

|  | **Individual cohorts** | | | **Studies combined** | | |
| --- | --- | --- | --- | --- | --- | --- |
|  | **n** | **HR** ^*^ **(95% CI)** | ***p*** |  | **HR** ^†^ **(95% CI)** | ***p*** |
| **RS-I** | 27 | **7.18** (2.53-20.4) | <0.001 |  | **6.22** (2.39-16.2) <0.001 | |
| **RS-II** | 05 | 2.98 (0.28-31.9) | 0.367 |  |  |  |

* Hazard ratios from Weibull models, expressed per 1 mg/dL (0.32 mmol/L) increase in phosphate

levels constrained to normal values (2.5-4.5 mg/dL; 0.81-1.45 mmol/L)

† Studies combined from meta-analyses using fixed-effect models

**Supplementary Table 6**. Serum phosphate levels and all-cause and CVD mortality in men from RS-I and RS-II stratified by smoking status, adjusted for age and BMI, follow-up until year 2012:

|  | **All-cause mortality** | | | **CVD mortality** | | |
| --- | --- | --- | --- | --- | --- | --- |
|  | **n** | **HR*(95% CI)** | ***p*** | **n** | **HR*(95% CI)** | ***P*** |
| **RS-I** |  |  |  |  |  |  |
| Never smoker | 57 | **2.11** (1.04-4.26) | 0.038 | 16 | 1.17 (0.28-4.88) | 0.827 |
| Former smoker | 543 | **1.66** (1.36-2.04) | <0.001 | 184 | **1.80** (1.28-2.52) | 0.001 |
| Current smoker | 210 | **1.36** (0.99-1.86) | 0.059 | 66 | **1.97** (1.11-3.51) | 0.021 |
| **RS-II** |  |  |  |  |  |  |
| Never smoker | 29 | 0.95 (0.37-2.47) | 0.923 | 08 | 1.45 (0.25-8.25) | 0.674 |
| Former smoker | 162 | 1.11 (0.76-1.63) | 0.570 | 47 | 1.65 (0.81-3.33) | 0.165 |
| Current smoker | 71 | 1.31 (0.76-2.26) | 0.331 | 22 | 0.74 (0.27-2.05) | 0.563 |
| **Studies combined** † |  |  |  |  |  |  |
| Never smoker | 86 | 1.59 (0.90-2.80) | 0.109 | 24 | 1.27 (0.42-3.85) | 0.667 |
| Former smoker | 705 | **1.52** (1.27-1.82) | <0.001 | 231 | **1.77** (1.30-2.40) | <0.001 |
| Current smoker | 281 | **1.35** (1.02-1.77) | 0.032 | 88 | 1.55 (0.94-2.56) | 0.085 |

* Hazard ratios from Weibull models, expressed per 1 mg/dL (0.32 mmol/L) increase in

phosphate levels

† Studies combined from meta-analyses using fixed-effect models

**Supplementary Table 7**. Serum phosphate levels and COPD mortality in men from RS-I stratified by smoking status, adjusted for age and BMI, follow-up until year 2012:

|  | **COPD mortality** | | |
| --- | --- | --- | --- |
|  | **n** | **HR* (95% CI)** | ***p*** |
| **RS-I** |  |  |  |
| Never smoker | 01 | n/a |  |
| Former smoker | 20 | **5.59** (2.21-14.1) | <0.001 |
| Current smoker | 07 | 2.64 (0.38-18.3) | 0.326 |

* Hazard ratios from Weibull models, expressed per 1 mg/dL

(0.32 mmol/L) increase in phosphate levels
